# Cell-mediated immune resistance in cancer

**DOI:** 10.20517/cdr.2019.98

**Published:** 2020-01-02

**Authors:** Yuhao Wang, Emily Hays, Martina Rama, Benjamin Bonavida

**Affiliations:** Department of Microbiology, Immunology, and Molecular Genetics, David Geffen School of Medicine, The University of California, Los Angeles, Los Angeles, CA 90025-1747, USA.

**Keywords:** Immune system, immune resistance, survival pathways, heterogeneity, cancer, T cells, checkpoint inhibitors, tumor microenvironment

## Abstract

The genetic and epigenetic aberrations that underlie immune resistance lead to tumors that are refractory to clinically established and experimental immunotherapies, including monoclonal antibodies and T cell-based therapies. From various forms of cytotoxic T cells to small molecule inhibitors that revamp the tumor microenvironment, these therapies have demonstrated notable responses in cancer models and a resistant subset of cancer patients, used both alone and in combination. However, even current approaches, such as those targeting checkpoint molecules, tumor ligands, and involving gene-related therapies, present a challenge in non-responding patients. In this perspective, we discuss the most common mechanisms of immune resistance, including tumor heterogeneity, tumor ligand and major histocompatibility complex modulation, anti-apoptotic pathways, checkpoint inhibitory ligands, immunosuppressive cells and factors in the tumor microenvironment, and activation-induced cell death. In addition, we discuss the strategies designed to circumvent these resistance pathways to showcase the potential of emerging technologies in battling the rise of resistance.

## INTRODUCTION

Immunotherapies have joined the ranks of conventional cancer treatments, such as radiation, chemotherapy, and surgery, and hold promise in extending the patient survival in multiple tumor types. Researchers in the field have scrambled to harness the natural cytotoxicity mediated by innate cells and peripheral blood lymphocytes^[1]^. High levels of cytotoxic T cell activation and expansion are good indicators of clinical response and tumor regression, whereas patients with tumors facing little resistance from the endogenous immune system are at heightened risk of cancer progression^[2,3]^. Despite our broadening knowledge, few strategies have been comprehensive enough to prevent or circumvent the waning effectiveness of the immune system against resistant cancer cells.

Modern approaches exploit a litany of mechanisms that differentiate aberrant cells from normal cells, seeking to balance the effectiveness of the treatment with its toxicity to the patient. However, aggressive tumors and late stage cancers display underwhelming responses to standard immunotherapies due to biochemical barriers, including decreased immunogenicity and increased immune tolerance^[4]^. In most cases, tumor cells develop the means to escape detection and/or killing by effector cells through a process of selection. Throughout the lifetime of a tumor, immunologically “hot” tumors - defined by an inflamed phenotype characterized by high intratumoral T cell activity - can adapt to become “cold” or immunosuppressive, which causes the tumor to relapse^[5]^. A poor understanding of these challenges threatens to stall the development of targeted anti-cancer immunotherapies and their translation into the clinic.

Multiple mechanisms of cancer immune resistance have been proposed over the last few decades: tumor heterogeneity, tumor antigen and major histocompatibility complex (MHC) modulation, anti-apoptotic pathways, checkpoint inhibitory ligands, immunosuppressive cell subsets, immunosuppressive factors in the tumor microenvironment (TME), and T cell activation-induced cell death (AICD). This perspective focuses on providing background for each of these mechanisms as well as summarizing the recent approaches to overcome resistance, including chimeric antigen receptor (CAR) T cells, checkpoint inhibitors, immune-sensitizing agents, TME-targeting strategies, and combination therapies.

## IMMUNE RESPONSE TO CANCER

### Innate immune response

Dendritic cells (DCs), natural killer (NK) cells, and macrophages comprise the most well-studied cell types of the innate immunity. While the majority of their activities are aimed toward eliminating unwelcome pathogens, they also bridge the short-lived innate response with the long-lasting adaptive response through functions ranging from cytokine and chemokine secretion to antigen presentation.

DCs represent the most essential professional antigen presenting cells (APCs), well known for their contributions to initiating cell-mediated immunity^[6,7]^. DCs prime tumor-specific CD4+ and CD8+ T cells by presenting three signals necessary for complete T cell activation: (1) peptide-MHC class I/II complexes; (2) costimulatory molecules CD80/CD86; and (3) cytokines that induce T cell proliferation and differentiation^[8]^. Their role during tumor rejection is derived from the activation of pattern recognition receptors (e.g., Toll-like receptors and the C-type lectins) which respond to a vast array of tumor-derived intracellular molecules known as damage-associated molecular patterns (DAMPs) released by cells undergoing immunogenic cell death and necrosis^[9,10]^. DCs can also sense engulfed tumor-derived DNA via the stimulator of the interferon genes complex pathway, which induces the production of type I interferons, stimulating DCs to uptake, process, and present tumor-associated antigens on MHC molecules^[3]^. DAMPs can also induce tumor-infiltrating DCs to phagocytose apoptotic cancer cells via endocytic and scavenger receptors^[11]^, further facilitating antigen presentation and solicitation of tumor-specific cytotoxic T lymphocytes (CTLs)^[12]^.

NK cells are innate lymphoid cells that form the first line of defense against infection, cancer, and metastasis using a special method of differentiating normal from aberrant cells^[13]^. Unlike T and B cells, NK cells lack receptors of antigen specificity; thus, rather than rely on tumor-associated antigens, NK cells pursue cells based on the integration of positive and negative signals received by activating and inhibitory receptors, whereby a decision is made on the relative balance of the signals as to whether the cell remains inactive or initiates killing^[13,14]^. Activating receptors [e.g., NK group 2D (NKG2D) and natural cytotoxicity receptors] recognize ligands highly expressed on tumor cells but deficient on normal cells, whereas inhibitory receptors recognize MHC class I molecules and other non-classical MHC molecules, which are frequently downregulated in tumor cells^[15]^.

Once activated, the NK cell mediates cell cytotoxicity through the release of granules containing perforin/granzyme and the ligation of Fas ligand (FasL) and tumor necrosis factor (TNF)-related apoptosis-inducing ligand (TRAIL) to their corresponding tumor cell receptors Fas and death receptors 4 (DR4) and 5 (DR5), respectively^[16]^. A crosstalk between NK cells and B and T cells occurs through activating Fcγ receptors that mediate antibody-dependent cell cytotoxicity (ADCC)^[17]^ and through the secretion of pro-inflammatory mediators [e.g., interferon γ (IFN-γ) and tumor necrosis factor α (TNF-α)] that shape T cell responses and recruit other immune cells to the inflamed tissues^[18]^. Abundant evidence has linked the prevalence of NK cells in tumors to its role in inhibiting tumor progression. For example, higher levels of NK cells in squamous cell carcinoma and resectable pancreatic cancer patients may delay tumor recurrence, increase patient survival, and prevent metastasis^[19,20]^.

Tumor associated macrophages (TAMs) diverge into functional phenotypes when encountering hypoxic environments, nutrient deficiency, and the host of soluble factors released by stromal cells^[21]^. Macrophages exist on a continuum of activation states - though grouping macrophages into either M1 or M2 phenotypes based on their secretory profiles remains common practice^[21,22]^. M1 “killer” populations produce ROS, inducible nitric oxide synthase, inflammatory cytokines (e.g., IL-1, IL-6, IL-12, IL-23, and TNF), and express MHC class II molecules; M2 “alternatively activated” populations produce transforming growth factor-β (TGF-β), arginase 1 (ARG1), and IL10^[22,23]^. Similar to DCs, macrophages are APCs that phagocytose roaming pathogens and fragments of apoptotic cells to present their antigens on MHC class I and II molecules^[11]^. The tumoricidal M1 macrophages release pro-inflammatory factors that induce tumor-rejecting Th1 responses, characterized by cell-mediated immunity. Lung tumors enriched with M1 macrophages experience decreased cell viability, growth, and angiogenesis with an increased sensitivity to chemotherapeutics such as cisplatin^[24]^. Ovarian cancer patients tend to exhibit better prognoses with higher M1 to M2 ratios^[23]^; the latter subset of immunosuppressive macrophages is discussed in the “Immunosuppressive Cell Subsets” section.

### Adaptive immune response

The adaptive arm of the immune system stands at the forefront of many of the immune therapeutics currently available^[25]^. CD4+ T helper lymphocytes (Th), CD8+ CTLs, and B cells each play a role in coordinating an initial primary and a long-term memory anti-tumor response that destroy emerging lesions. This process of immune editing consists of three phases: elimination, i.e., the tumor-killing response mounted by the immune system; equilibrium, i.e., the period of selection for non-immunogenic phenotypes; and escape, i.e., the phase when the resistant cells rapidly multiply to form a malignancy^[26]^.

Naïve T cells require two signals to mature into effector T cells: (1) crosslinking of the T cell receptor (TCR) with the major histocompatibility complex (MHC; class I for CTLs and class II for Th cells); and (2) binding of CD28 to CD80/CD86 (B7 ligands) on APCs. The costimulatory signal is key to T cell activation as it prevents a state of growth arrest (anergy) from TCR-MHC binding^[27]^. True to their name, the Th cells participate in the efficiency of CTL activation and memory induction by presenting costimulatory molecules and cytokines such as IL-2 and IFN-γ, which upregulate the expression of MHC class I molecules on APCs^[28]^.

T cell-driven inflammation in intratumoral compartments is largely responsible for cell-mediated anti-tumor activities. Haabeth *et al*.^[29]^ proposed a tumor-specific role for the inflammatory Th1 response produced in part by the secretion of pro-inflammatory cytokines IL-1, IL-6, TNF-α, and IFN-γ by Th1 cells as opposed to the immunosuppressive Th2 response^[30]^. Typical αβ T cell-mediated cytotoxicity depends on the release of perforin/granzymes and on the expression of surface death ligands upon detection of a target antigen loaded on MHC class I molecules^[31]^. The more unconventional γδ T cells (named after their distinctive TCR chains) are not restricted to killing in the presence of MHC molecules but also through NKG2D and CD16^[32,33]^. In addition to their antigen specificity γδ T cells exhibit myeloid cell-like qualities, having the ability to phagocytose protein antigens and present both antigens and costimulatory signals in the style of the professional APCs^[34,35]^.

Lastly, B cells are important for their ability to produce antibodies and for their immune regulatory functions, such as antigen presentation, costimulation, and cytokine secretion. B cells play into a dichotomy of roles in tumorigenesis, at once producing immunosuppressive cytokines and inducing antibody-directed cell cytotoxicity through the formation of antibody-antigen complexes depending on the tumor type and conditions in the TME^[36]^. Monoclonal antibodies have been used extensively in cancer immunotherapy for their diversity and specificity generated through somatic recombination. The list of FDA-approved antibodies for cancer therapy continues to expand, including ones against cell surface antigens, such as human epidermal growth factor receptor 2 (HER2), programmed-death ligand 1 (PD-L1), cytotoxic T-lymphocyte-associated protein 4 (CTLA-4), and CD20, as well as soluble proteins, such as vascular endothelial growth factor (VEGF) and TNF^[37]^.

## MECHANISMS OF IMMUNE RESISTANCE

**Figure 1.**
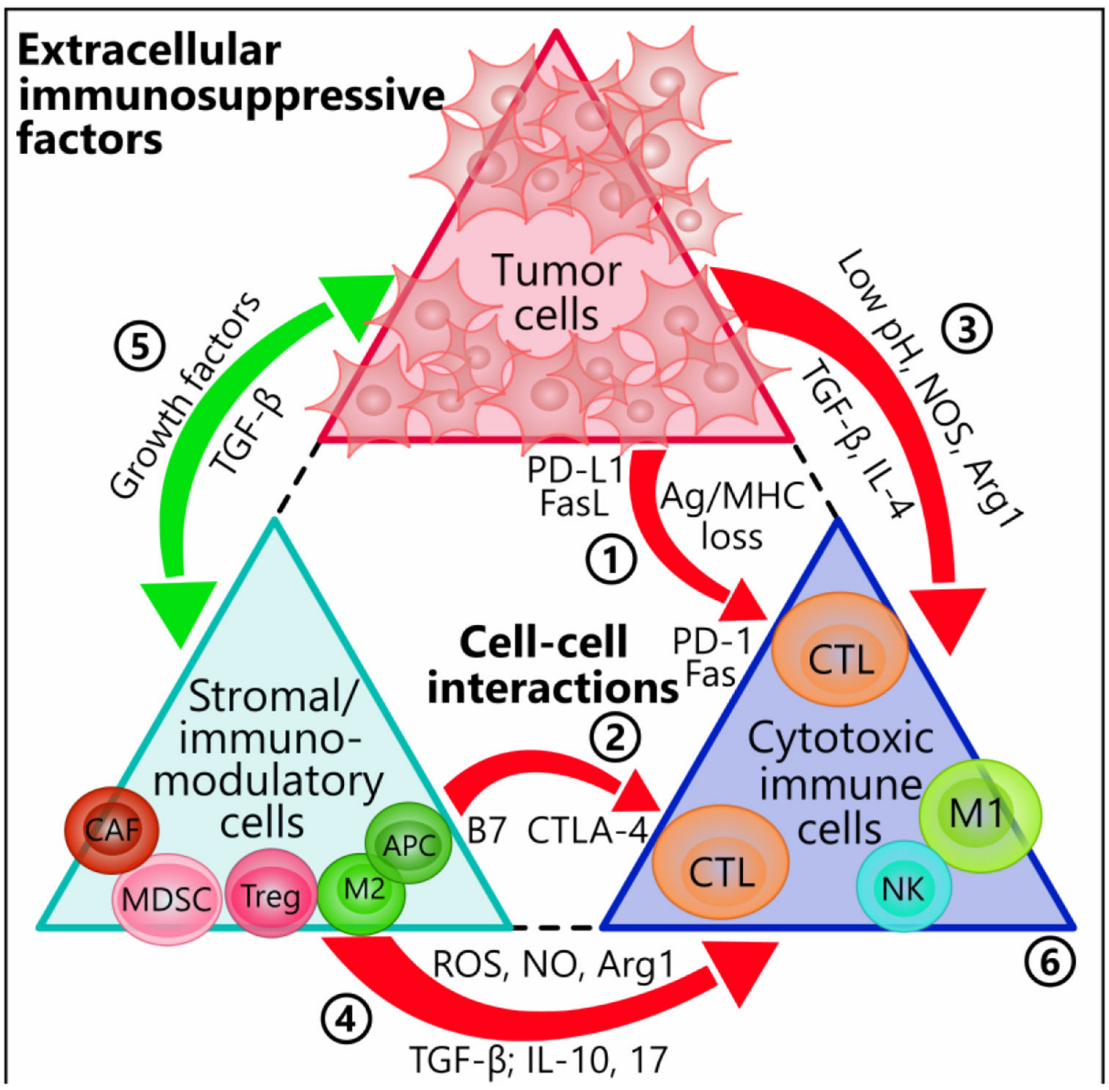
Tumor- and stromal cell-mediated immunosuppressive interactions. Both tumor cells and the surrounding stromal cells heavily influence the tumor-specific cytotoxic immune cells via direct cell-cell contact and paracrine factors. Red arrows denote negative, or inhibiting, influences and green arrows positive, or activating, influences. (1) Tumor cells increase the expression levels of checkpoint molecules such as PD-L1 and FasL that attach to CTLs expressing PD-1 and Fas, respectively. Tumors may also escape detection altogether from attackers by modulating MHC and antigenic levels. (2) B7 ligands on APCs interact with CTLA-4 on T cells to inhibit the latter’s activities. (3) Tumor cells modify the TME by producing excess acidic metabolites, NOS, Arg1, and the immunosuppressive cytokines TGF-β and IL-4. (4) Likewise, the tumor stroma shares in these activities by producing similar factors as well as ROS, IL-10, and IL-17. (5) The sharing of growth factors and pro-tumor cytokines between tumor cells and stromal cells further amplifies the inhibitory effect on anti-tumor immune cells. (6) Depending on the mechanisms encountered, the anti-tumor immune cells can undergo multiple pathways of inhibition, including exhaustion, lack of tumor targeting, or apoptosis. Arg1: arginase 1; CAF: cancer-associated fibroblast; CTL: cytotoxic T lymphocyte (CD8+); CTLA-4: cytotoxic T-lymphocyte-associated protein 4; DC: dendritic cell; FasL: fas ligand; M1: M1 macrophage; M2: M2 macrophage; MDSC: myeloid-derived suppressor cell; MHC: major histocompatibility complex; NOS: nitric oxide synthase; NK: natural killer; PD-1: programmed cell death protein 1; PD-L1: programmed death-ligand 1; ROS: reactive oxygen species; TGF-β: transforming growth factor-β; TME: tumor microenvironment; Treg: T regulatory cell; APCs: antigen presenting cells

A wide variety of factors and interactions underlie the phenomenon of immune resistance in cancers. We outline these mechanisms in the following sections and in Figure 1.

### Tumor cell heterogeneity

Variations in the cancer patient survival after treatment highlight the bewildering heterogeneity both between and within tumors. Tumor composition is wildly complex, often containing diverse populations of tumor cells with a wide range of genetic and epigenetic differences^[38]^. Interpatient heterogeneity denotes the phenomenon that no two patients with the same type of cancer will respond to a given treatment, nor progress, in the same manner^[39]^. Intratumoral heterogeneity assumes a plurality of lineages selected for by cancer treatment or the endogenous immune system. Subclones with acquired resistance mechanisms and self-renewing properties often survive treatment and lay quiescent until the selective pressure is removed^[40]^. Such cells can develop the ability to produce signaling factors that increase the tumorigenicity and growth capacity of neighboring tumor cells in a process called subclonal cooperativity^[38]^, suggesting tumor cell adaptation to be less an isolated than a local and sometimes global event.

Intratumoral heterogeneity [both spatial (different localities) and temporal (over time)] remains a constant threat to immunotherapies. Tumor cells, boasting an inherent plasticity, undergo a continuous process of Darwinian co-evolution with each other and with the host immune cells^[40]^. Tumor cells are known to escape from immune surveillance via modulation of their antigenic landscapes and expression of checkpoint inhibitory ligands^[41]^. The cytokine content in the TME can also modify tumor cell differentiation and T cell resistance^[42]^. The pro-tumorigenic feedback loops that are generated from the widening influence of resistant subclones underlie the limited efficacy of current immunotherapies. However, high mutational load and expanded sets of exploitable neo-antigens in certain cancers may correlate to overall survival^[39,43]^. Current strategies to analyze the aspects of immune resistance include interdisciplinary frameworks that use phylogenetic analyses to reconstruct a tumor’s history and evolutionary trajectories^[38,44]^. Understanding the selective processes that cause tumor progression will guide the field toward insights on how to prevent tumor relapses and enhance treatment efficacy.

### Tumor cell modulation of surface antigens and MHC molecules

Early reports have described the association between alterations in tumor ligand presentation and tumor progression. Cervical carcinoma and melanoma cells lose expression of MHC class I molecules and tumor antigens to evade detection by immune cells with both events observed to occur simultaneously as a result of ongoing immune pressure^[45-47]^. Recent studies corroborate the observation that T cells select for tumor cell variants with reduced or lost neo-antigen expression^[48]^. Tumors losing MHC class I expression have low immunogenicity and generally exclude tumor-infiltrating lymphocytes to the peritumoral regions^[49]^. Loss of MHC class II molecules in diffuse large B cell lymphoma cells and in microglia responding to gliomas promotes immune evasion by affecting the priming of APCs and CD4+ Th cells^[50,51]^.

Loss of the MHC class I and II molecules occurs via various genetic and epigenetic mechanisms. The human MHC or human leukocyte antigen class 1 (HLA-I) molecules are encoded by a series of polymorphic genes with multiple alleles or haplotypes. Alterations to these alleles are either reversible (soft) or irreversible (hard) with multiple possible HLA-I phenotypes depending on the type of alteration^[49]^. The most common method is the loss of heterozygosity in the HLA and β2-microglobulin (an essential factor of the HLA-I complex)-encoding regions of chromosomes 6 and 15, resulting in HLA-I haplotype loss^[49]^. Total loss of HLA-I expression may occur in colorectal cancers through either β2-microglobulin inactivation or downregulation of the antigen presentation machinery LMP7/TAP2^[47,52]^, potentially induced by higher DNA methylation levels of the associated genes^[53]^. Mouse models have implicated the role of the mitogen-activated protein kinase (MAPK)/extracellular signal-regulated kinase (ERK) pathway in the epigenetic control of MHC class II expression^[50]^. Glioma cells expressing Toll-like receptor 2 (TLR2) ligands (e.g., heat shock proteins and extracellular matrix components) trigger the activation of microglial TLR2 and signaling through MAPK/ERK, leading to the deacetylation of histone H3K9. This inhibits the expression of *Ciita*, a gene for the master coactivator of MHC class II expression, which leads to low MHC class II presentation on microglial cells.

Tumor cells also have the propensity to evade detection by NK cells by modulating the expressions of the MHC class I-like NKG2D ligands, which bind to the activating NKG2D receptors on NK cells to trigger tumor cell lysis^[54]^. Post-translational regulation of these ligands occurs via intracellular retention^[55]^, internalization and proteasomal degradation^[56]^, and cleavage and shedding^[57]^, each leading to inhibition of NKG2D-mediated cell targeting by NK cells.

### Anti-apoptotic mechanisms

Tumor cells contain intricate networks of hyper-activated signaling pathways that protect the cell from apoptosis and ensure their continued survival and proliferation. A common oncogenic variant of BRAF (BRAF^V600E^) in melanomas potently activates the RAS/BRAF/MEK/ERK (MAPK) pathway, which promotes tumor immune editing, the expression of immunosuppressive cytokines and checkpoint markers, and reduces CTL infiltration^[58,59]^. The hyper-activated phosphoinositide 3-kinase (PI3K)/Akt pathway shares these functions following the loss of its physiological inhibitor, the phosphatase and tensin homolog (PTEN)^[60]^. Aberrant PI3K/Akt pathway signaling also inhibits apoptosis by upregulating the expression of B cell lymphoma 2 (Bcl-2) and decreasing the levels of apoptosis regulators such as p53 and the Bcl-2-associated X^[61,62]^. Bcl-2 protects cancer cells against CTL-mediated cytotoxicity and is under the regulation of several oncogenic pathways, including the MAPK pathway^[63,64]^. Researchers in the past have identified Bcl-2 as a predictive marker of patient response to immunotherapy in clinical samples of metastatic renal cell carcinoma^[65]^, raising the possibility of targeting Bcl-2 to enhance the vulnerability of cancer cells to immunotherapies^[66]^.

A major mode of extrinsically-activated apoptosis is triggered by the activation of death receptors on tumor cells. NK cells, monocytes, and DCs^[67,68]^ express the tumoricidal cytokine TRAIL, which binds to DR4 and DR5 to induce the formation of the death-inducing signaling complex (DISC) and trigger caspase-dependent cell death^[69,70]^. Previous reports have shown that tumor cells become refractory to TRAIL-mediated apoptosis upon downregulating DR5 via the transcriptional repressor yin yang 1 (YY1)^[71,72]^. The master anti-apoptotic regulator cellular FLICE (FADD-like IL-1β-converting enzyme)-inhibitory protein (c-FLIP) evokes the same effect in tumor cells. c-FLIP can interfere with the formation of the DISC in HER2-positive breast cancer and human osteogenic sarcoma cells by binding to the death receptor adaptor FADD and caspases 8 and 10^[69,73]^. A cleavage product of c-FLIP (p22-FLIP) can also activate the cytoprotective nuclear factor κB (NF-κB) pathway in malignant cells, further reducing the potency of TRAIL-mediated apoptosis^[74]^.

### Immune checkpoint ligands

Coinhibitory checkpoint ligands on an APC can interact with receptors on a lymphocyte to guard against autoreactivity and maintain peripheral tolerance. T cells undergo anergy or exhaustion (“off switch”) when their programmed death receptors encounter these checkpoint ligands and become unable to mount a complete response against their target^[75]^. Although a repertoire of such ligands is characteristic of normal tissues, tumor cells are known to coopt this ability to suppress tumor-specific T cell function^[75,76]^. PD-L1 and CTLA-4 are among the most well-studied checkpoint ligands, although other inhibitory molecules include T-cell immunoglobulin and mucin-domain containing-3 (TIM-3), lymphocyte-activation gene 3 (LAG3), and indoleamine 2,3-dioxygenase (IDO)^[76]^.

Researchers have made a significant effort to understand how cancer cells harness the programmed cell death protein 1 (PD-1): PD-L1/2 pathway to evade the host immune response. Signaling through PD-1 in T cells subverts TCR signal transduction and CD80/CD86-CD28 costimulation, inhibiting cytokine release and causing cell cycle arrest^[77]^. While the exact mechanisms of how PD-L1 is upregulated in cancer cells remain unclear, previous evidence suggests that the transcription factor YY1^[78]^, phosphatases Src homology region 2 domain-containing phosphatase-1 and 2 (SHP 1/2)^[77]^, and cyclin-dependent kinases 4 and 6 (CDK 4/6)^[79]^ all regulate PD-L1 expression in some way. PD-L1 expression in various cancers, such as lung cancer, colorectal cancer, and melanoma, has been correlated with cancer progression and poor survival^[80]^ and dampened signs of CTL activity^[75]^.

CTLA-4 expression is induced upon TCR-CD28 costimulation^[81]^ and blocks T cell activation when engaging CD80/CD86 on the surfaces of APCs^[76]^. Since the majority of CTLA-4 is localized to intracellular compartments, surface expression of CTLA-4 relies primarily on exocytosis controlled by phospholipase D, GTPases, and the TCR-interacting molecule^[82,83]^. Several mechanisms of CTLA-4-mediated T cell downregulation have been proposed. For example, researchers have shown that CTLA-4 outcompetes CD28 in binding CD80/CD86, thus preventing T cell costimulatory activation by CD28^[76,84]^. CTLA-4 can also block T-cell activation through the activation of protein phosphatases^[81]^ and by removing CD80/CD86 from the surface of APCs^[85]^. DCs cocultured with CTLA-4+ breast cancer cells expressed lower amounts of HLA-DR and costimulatory molecules, inhibited CD4+ and CD8+ T cell differentiation, and facilitated tumor growth^[86]^. Regulatory T (T_reg_) cells also constitutively express CTLA-4, which can decrease APC potency in activating conventional T cells^[87]^.

### Immunosuppressive cell subsets

The “congenial soil” hypothesis describes an advantageous growth environment for primary and metastatic tumor cells^[88]^. The discussion of the TME as a therapeutic target begins with understanding the diverse set of infiltrating immune cells that differ in activation status and identity based on the tumor characteristics. T_reg_ cells, myeloid-derived suppressor cells (MDSCs), and M2 macrophages represent the some of the most prevalent immunosuppressive cells in the TME and in the peripheral circulation.

T_reg_ cells are CD4+CD25+ (as well as CD8+^[89,90]^) T cells that express the master T_reg_ transcription factor forkhead box P3 (FOXP3)^[91,92]^. CD4+ T_reg_ cells phenotypically diverge into two main subsets: CD4+CD45RA+FOXP3^1ow^ naïve cells with weakly suppressive functions and CD4+CD45RA−FOXP3^hlgh^ effector cells with strongly suppressive functions^[93]^. Since T_reg_ cells are essential in preventing autoimmunity, it can be difficult to define the balance between “good” and “bad” T_reg_ activities when considering depletion as a therapeutic option^[92]^. However, T_reg_ cells are widely considered to be pro-tumorigenic, being armed with myriad factors that inhibit CD4+ and CD8+ T cell proliferation and effector functions, including inhibitory cytokines (IL-10, TGF-β), immune checkpoint ligands^[94,95]^, and granzyme/perforin to induce cell death in NK cells and CTLs^[96]^. Clinically, high rates of intratumoral T_reg_ infiltration are detrimental to patient survival and beneficial to tumor growth in cancers such as gastric cancer^[97]^ and ovarian cancer^[98]^.

The MDSCs compose a set of immature myeloid cells abundant in tumors and peripheral lymphoid organs with varying functions depending on their local tissue compartment^[99]^. MDSC markers include CD11b and Gr-1 with CD11b+Ly6C^low^Ly6G^high^ cells belonging to the granulocytic subset and CD11b+Ly6C^high^Ly6G^low^ belonging to the monocytic subset^[100]^. MDSCs are typically induced by tumor-derived pro-inflammatory mediators and thus contribute to an inflammatory response in the tumor milieu^[101]^. MDSCs possess dual functionality as immunosuppressive cells: regulating both the non-immunological - tumor growth and metastasis - as well as the immunological aspects of tumorigenesis^[100]^. MDSCs suppress multiple types of immune effectors including T and NK cells by producing large quantities of reactive oxygen species (ROS), nitric oxide, arginase 1 (Arg1, which depletes arginine, an important metabolic molecule in T cells), and inhibitory cytokines^[101]^. They can disable naïve T and B cells by post-transcriptionally downregulating their expression of the L-selectin lymph node homing receptor, impairing immune cell activation and trafficking to distant lymph nodes^[102]^. Their influence in the immunosuppressive TME expands via crosstalk, with activities such as promoting the development of T_reg_ cells^[103]^, suppressing dendritic cell (DC) maturation^[101]^, and converting M1 macrophages to the M2 type^[104]^.

M2 macrophages (also TAMs) have anti-inflammatory qualities to protect host tissues and produce numerous growth factors, including epidermal growth factor, fibroblast growth factor, and VEGF that enhance tumor cell growth and vascularization^[23]^. Large quantities of M2 macrophages localized to the lungs are key to establishing an opportunistic microenvironment for metastatic breast cancer cells^[105]^. TAMs can bind to vascular cell adhesion 1 on the surfaces of the cancer cells to enhance cancer cell survival and resistance to death signals (e.g., TRAIL) by activating PI3K/Akt pathway signaling. Similar to MDSCs, M2 macrophages produce Arg1 to deplete L-arginine from the microenvironment, which enables them to impair T cell memory formation and survival^[106,107]^. They express IL-10, which negatively affects production of the CTL-stimulating IL-12 by DCs expressing IL-10 receptor^[108]^. Factors secreted from the local tumor stroma can induce the expression of PD-L1 on tumor-activated monocytes^[109,110]^, while M2-derived C-C motif chemokine ligand 22 (CCL22) can enhance T_reg_ cells trafficking into the intratumoral compartment^[98]^. These M2-related activities, as well as the bidirectional crosstalk with MDSCs, further broaden their role in increasing T cell anergy and sculpting the TME^[22]^.

### Suppressive factors in the TME

Tumor cells often produce immunosuppressive cytokines as a way to avoid the anti-tumor immune response. TGF-β is a paracrine signaling mediator with varied roles in cell proliferation, apoptosis, differentiation, metastasis, and immune regulation^[111]^. Tumors use TGF-β in indirect methods of immune evasion: converting CD4+CD25− to CD4+CD25+ T_reg_ cells^[112]^ or inducing the development of a Th17 type response designed to counter autoimmunity and reduce the fraction of activated Th cells^[113]^. In a mouse model of colorectal cancer, the typically immunologically “cold” tumors secreted TGF-β to knock down Th1 differentiation and T cell infiltration^[114]^. Tumor-activated stromal cells can also produce TGF-β, indicating the cyclical nature of TGF-β signaling within the TME. In metastatic urothelial cancer, TGF-β-producing stromal fibroblasts can shift the anti-tumor inflamed phenotype within the tumor to an excluded phenotype with CTLs secluded to the peritumoral regions^[115]^. Th2 cytokines, such as tumor-derived IL-4, can help generate M2 macrophages and MDSCs to block tumor-specific CTL activity^[116]^. Treating murine and human myeloid DCs with exogenous IL-10 increases PD-1 expression and amplifies production of IL-10, leading to suppressed T and B cell responses and acquisition of resistance to anti-PD-1 therapy by ovarian cancer cells^[117]^. The effects of other immunosuppressive cytokines, such as IL-17, in clinical tissues are well-documented and are associated with poor prognoses^[118]^.

Besides cytokines, several other tumor-associated mediators participate in the dynamic network of signals facilitating immune tolerance. In their comprehensive review, Junttila and De Sauvage^[119]^ outlined the roles of the cancer-associated fibroblasts (CAFs) in producing angiogenic factors and chemokines [granulocyte-macrophage colony-stimulating factor (GM-CSF) and granulocyte-colony stimulating factor (G-CSF)] to attract myeloid cells. Alternatively, chemokines can be modified to favor immune suppression; reactive nitrogen species and peroxynitrite generated by Arg1 and nitric oxide synthase (NOS) in tumor cells and MDSCs can nitrosylate C-C motif chemokine ligand (CCL2) - a major inflammatory chemoattractant for T cells, NK cells, and myeloid cells - to produce a modified form of CCL2 that recruits only myeloid cells^[120]^.

Some researchers have attempted to define stromal angiogenic/hypoxia-associated gene signatures in tumors and how they are associated with immune cell exclusion and immunosuppressive cell trafficking^[121]^. In practice, others have found that tumor hypoxia upregulates CCL28, a chemokine that recruits CD4+CD25+FOXP3+ T_reg_ cells in an ovarian cancer mouse model, linking it to a more tolerigenic TME and increased tumor vascularization via vascular endothelial growth factor A^[122]^. VEGF was previously shown to block immature myeloid cell and DC maturation, reducing their antigen presentation capabilities and dampening the immune stimulatory phenotype^[123]^. When tumors are poorly vascularized, acidification becomes a major concern due to inadequate blood flow and accumulation of acidic metabolites. T cells become anergic in the low pH (pH 6-6.5) of poorly perfused tumors, exhibiting impaired IFN-γ and TNF-α production^[124]^.

### AICD

AICD acts as a homeostatic “self-nonself discrimination” mechanism that coordinates programmed cell death of self-reactive lymphocytes^[125]^. T cell populations expand considerably upon antigenic stimulation, followed by a period of contraction as cells targeting self-antigens are deleted. Clonal deletion presents a technical barrier for adoptive cell therapies that use T cells with tumor antigen-specific TCRs or engineered TCRs by affecting both developing and mature T cells^[126,127]^.

AICD occurs through caspase-dependent and caspase-independent mechanisms. Caspase-dependent AICD relies on the interaction between Fas-expressing T cells and FasL-expressing tumor cells to trigger a caspase cleavage cascade within the T cell, leading to apoptosis^[128]^. Caspase-independent AICD occurs upon repetitive TCR stimulation and c-JUN N-terminal kinase activation, which triggers the release of mitochondria-derived death effectors^[126,127,129]^. p53 may also contribute to a mitochondria-centric cell death by activating a DNA damage response pathway consisting of DNA damage marker γH2AX upregulation and ATM activation^[127]^. A recent study has reported the importance of a long non-coding RNA (lncRNA), NF-κB-interacting lncRNA (NKILA), in inhibiting NF-κB activity and potentiating AICD in CTLs and Th1 cells^[130]^.

## MECHANISMS OF REVERSAL OF IMMUNE RESISTANCE

The various mechanisms of immune resistance pose a constant threat to the reliability of our current arsenal of immune therapeutics. It is important to revise how we mobilize the immune system against tumor cells with a more informed approach toward increasing the adaptability and specificity of cancer treatment. Here, we discuss some of the most promising modalities.

### CAR T cells

CAR T cells exhibit notable advantages over previous TCR-based T cell therapies by combining the specificity of antibodies with the robustness of CTLs. CARs contain an ectodomain consisting of a single-chain variable fragment (scFv) derived from monoclonal antibodies, a transmembrane domain, and an intracellular signaling endodomain^[131]^. Whereas first generation CARs possess only a primary stimulatory CD3ζ endodomain, later generations contain fused costimulatory signaling domains (e.g., CD28 or 4-1BB) that relax the requirement for independent costimulatory signals to fully activate the tumor-killing response^[132,133]^. CAR T cells can also recognize and eradicate tumor cells in a MHC-independent manner, which targets cells that have escaped immune detection by modulating MHC expression^[132]^. Fourth-generation CARs possess additional nuclear factor of activated T-cells (NFAT) transcription factor domains; upon CAR ligation, liberated NFAT domains translocate to the nucleus to upregulate cytokine production, thus mobilizing the endogenous immune system and enhancing the scope of tumor recognition and killing^[133]^. These fourth-generation cells, called T-cells redirected for universal cytokine-mediated killing (TRUCKs), release IL-12 to rescue anti-tumor immune cells and promote epitope spreading to further enhance tumor rejection^[134]^.

Studies on CAR T cell treatments have demonstrated robust anti-tumor activity with high complete remission rates in patients with acute lymphoblastic leukemia, diffuse large B cell lymphoma, and chronic lymphocytic leukemia^[134]^. The FDA has approved two second-generation CAR T cell therapies targeting CD19 (Yescarta and Kymriah) for treatment against non-Hodgkin lymphomas and acute lymphoblastic leukemia^[135]^. Efforts to expand the CAR T cell repertoire have led to trials featuring CARs directed toward B-cell mature antigen enriched on multiple myeloma cells^[136]^ and several other tumor antigens related to solid tumors^[137]^. Dual-signaling CARs contain two distinct CAR molecules with different scFv domains. This method splits the primary and costimulatory signals between the two receptors and refines the discriminatory power of single antigen approaches^[132,134]^. Another innovative design makes use of a synthetic Notch endodomain that, once activated, promotes expression of a secondary CAR molecule with the same functions as normal CARs^[138]^. The trend toward multiple modulatory receptors means to mitigate on-target/off-tumor effects resulting from unintended toxicities, such as cytokine release syndrome and neurological toxicity, on bystander cells expressing low levels of tumor antigens.

### Checkpoint inhibitors

In recent years, the FDA has approved a few checkpoint blocking antibodies known as checkpoint inhibitors, such as ipilimumab targeting the CTLA-4 and nivolumab targeting the PD-1, in hopes of producing better clinical outcomes. These inhibitors have been shown to be effective in “flipping the switch” in many patients with PD-1+ and programmed-death ligand 1+ (PD-L1+) cancers^[139]^ with potential for therapeutic use in patients with metastatic sarcoma^[140]^, Hodgkin’s lymphoma^[141]^, and gastroesophageal cancer^[142]^. A phase 1 clinical trial testing nivolumab response in 23 patients with relapsed or refractory Hodgkin’s lymphoma observed a complete response in four (17%) patients and partial response in 16 (70%)^[143]^. Similarly optimistic results were shown in a phase 3 efficacy trial of adjuvant nivolumab against resected stage III or IV melanoma; however, the benefits observed with nivolumab were more significant than with ipilimumab (anti-CTLA-4), with additional concerns about the toxicity of the latter^[144]^. Despite this shortfall, it appeared that dual treatment with nivolumab and ipilimumab in metastatic melanoma may hold more promise than either treatment alone^[145]^.

The mechanisms by which checkpoint inhibitors stimulate the anti-tumor response are not completely known, although the prevailing theory seems to be a revitalization of T cell activity. Numerous studies (reviewed in^[146]^) have observed signs of improved anti-tumor responses including increased T cell activation markers, CTL infiltration, and pro-inflammatory cytokine/chemokine production. Metabolically, competition between tumors and T cells for glucose restricts T cell activity, whereas treatment with checkpoint inhibitors lowers glycolytic capacity in tumors, increasing the availability of glucose for T cells^[147]^. Optimistic strides aside, these drugs are not always effective and have been shown to drive resistance in up to 60% of patients treated^[148]^. Thus, researchers are beginning to pay special attention to the dosing effects, biodistribution, route of administration, and predictive biomarkers in different tumor types^[142,149-151]^ to develop additional strategies for improving checkpoint blockade therapy^[152]^.

### Immune-sensitizing agents

Many of the prominent oncogenic pathways lend well to targeting in anti-cancer therapies, and researchers in the field have zeroed in on promising inhibitory molecules that play double duty in the reversal of immune resistance. For example, inhibitors of YY1^[153]^ were shown to increase the sensitization of human prostate carcinoma cells to chemotherapeutic drugs and to FasL and TRAIL-mediated apoptosis^[71,154]^. MEK inhibitors against relapsed melanomas that exhibit resistance to BRAF inhibitors can rescue melanoma antigen expression and a tumor-rejecting TME^[59]^. However, this study also reveals the risk of single target approaches in selecting for resistant tumor cells; thus, current research seeks to inhibit parallel survival pathways by targeting anti-apoptotic proteins such as the X-linked inhibitor of apoptosis (XIAP), the cellular inhibitor of apoptosis protein 1/2 (cIAP1/2), and survivin to sensitize cancer cells to chemotherapeutics, antibody based-therapies, and TRAIL therapy^[155]^. Other small molecule inhibitors either in testing or on the market inhibit anti-apoptotic pathway proteins such as the Bcl-2 family of proteins^[156]^. Scientists testing a dual-functional vector encoding an shRNA to silence Bcl-2 and an ssRNA to stimulate immune cells observed increased gastric carcinoma cell apoptosis and suppressed proliferation^[157]^. Such approaches that strip the tumor cell of its defenses and prime the attacking immune cells can be used to tip the balance between immune sensitivity and resistance.

Other methods target the upregulation of tumor suppressors that exert suppressive activity on the same, if not wider, range of pro-tumorigenic molecules. Some drugs, such as ReACp53, block p53 inhibition and restore its tumor suppressor activity^[158]^. The use of the anti-CD20 monoclonal antibody LFB-R603 in non-Hodgkin’s lymphoma cells upregulates the tumor suppressors Raf-1 kinase inhibitory protein (RKIP) and PTEN^[159]^. Maintenance of RKIP expression prolongs its inhibition of the oncogenes nuclear factor κB (NF-κB) and Snail, resulting in sensitization of the cells to TRAIL. Xanthohumol, a prenylated chalcone found in hops, was effective in upregulating DR5 expression in neuroblastoma cell lines, enhancing TRAIL-mediated apoptosis and reducing cancer cell viability^[70]^.

### Targeting the TME

Whether therapies are directed to the cellular or the humoral aspect, targeting the TME takes advantage of the complex interconnectedness of the tumor ecosystem to sculpt a more immune-friendly phenotype^[160]^. Chronic inflammation in the TME presents a barrier to immunotherapies reinforced by the immunosuppressive cells recruited to the tumor^[161]^. Previous evidence suggested that depleting T_reg_ cells using antibodies against T_reg_ markers (CTLA-4 and OX40) at a single tumor site could generate a systemic anti-tumor response that eliminated disseminated metastatic tumor cells^[162]^. T_reg_ depletion in an aggressive subtype of triple-negative breast cancer was also effective at sensitizing tumors to anti-PD-1 therapy^[163]^. An agonistic TRAIL receptor 2 antibody reduced numbers of MDSCs in the peripheral blood and tumors of patients enrolled in a phase 1 trial, in which researchers established an inverse correlation between decrease in MDSCs and progression-free survival^[164]^. Targeting TAMs with anti-MARCO (suppressive TAM marker) antibody converted them to a pro-inflammatory phenotype that favored anti-tumor activity in multiple tumor types^[165]^, adding another entry to the arsenal of therapies against TAMs in restructuring the immune response^[166]^.

Alternative approaches aim to alter the TME on a more metabolic and angiogenic level. Cancer cells reside in harsh, nutrient-poor microenvironments, but stromal cells such as the CAFs can adapt to supplement the metabolic needs of the tumor. Yang and colleagues showed that CAFs exhibited an abnormal ability to synthesize glutamine for tumor cells in a glutamine-scarce microenvironment and that inhibiting this pathway reduced tumor weight and metastasis^[167]^. While clinical applications of angiogenesis inhibitors have revealed challenges of their own, anti-angiogenic drugs have proven effective in both the laboratory and the clinic (reviewed in^[168]^). One of these challenges is the enabling of tumor hypoxia and immune resistance, but certain strategies, such as hyperoxic conditioning, could reverse hypoxia-induced immune suppression and strengthen T and NK cell anti-tumor functions^[169,170]^.

### Combination therapies

The allure of combining different therapies stems from the prospect that targeting distinct molecular pathways or using different modalities minimizes the occurrence of resistance. Combination therapies often produce additive or synergistic effects, and many studies have described the potential in utilizing different permutations of radiation, vaccines, antibodies, and chemotherapeutics. Fu *et al*.^[171]^ noticed complete tumor regression in a mouse B16 melanoma model when treating mice with a cancer vaccine TEGVAX (consisting of GM-CSF and TLR agonists) and anti-PD-1 antibodies after observing that TEGVAX alone upregulated PD-L1 expression in the TME. Checkpoint inhibitors applied concurrently with stereotactic radiosurgery (a type of targeted, non-surgical radiation procedure) could improve overall survival and decrease incidence of brain metastases in patients with non-small cell lung cancer, melanoma, or renal cell carcinoma compared to radiosurgery alone^[172]^.

In some cases, researchers have acted on observations that the TME restructures itself as a response to single therapies. Mariathasan *et al*.^[115]^ combined anti-PD-L1 antibodies with TGF-β neutralizing antibodies to test the effect of knocking down immunosuppressive factors from CAFs on anti-PD-L1 therapy; they noticed significant reductions in tumor size in their model of metastatic urothelial cancer. Suppressing MDSCs using multikinase inhibitors and checkpoint inhibitors elicited a synergistic anti-tumor response against castration-resistant prostate cancer cells^[173]^. Pallasch *et al*.^[174]^ observed a synergistic response when treating a mouse model of acute lymphoblastic leukemia with cyclophosphamide and antibody-directed therapy (anti-CD52 and anti-CD20), resulting in almost total reduction of tumor burden in the bone marrow. They found that chemotherapeutic treatment increased macrophage involvement in the bone marrow, increasing phagocytic activity and induction of stress-related cytokines, effectively sensitizing the TME.

Conversely, adoptive cell transfer and engineered T cell therapies may benefit from suppression of AICD, but the possibility remains poorly explored. Huang *et al*.^[130]^ proposed inhibiting the lncRNA NKILA, which increases AICD in tumor-targeting T cells, using gene-editing or delivered shRNA as a potential therapeutic strategy to rescue adoptively transferred T cells from AICD and enhance CTL infiltration. Cao *et al*.^[175]^ reported that histone deacetylase inhibitors could inhibit FasL-mediated AICD in tumor-resident CD4+ T cells. Together, these results hold promise in combination with other anti-tumor therapies, especially those that rely on T cells.

## CLINICAL APPLICATIONS

**Figure 2.**
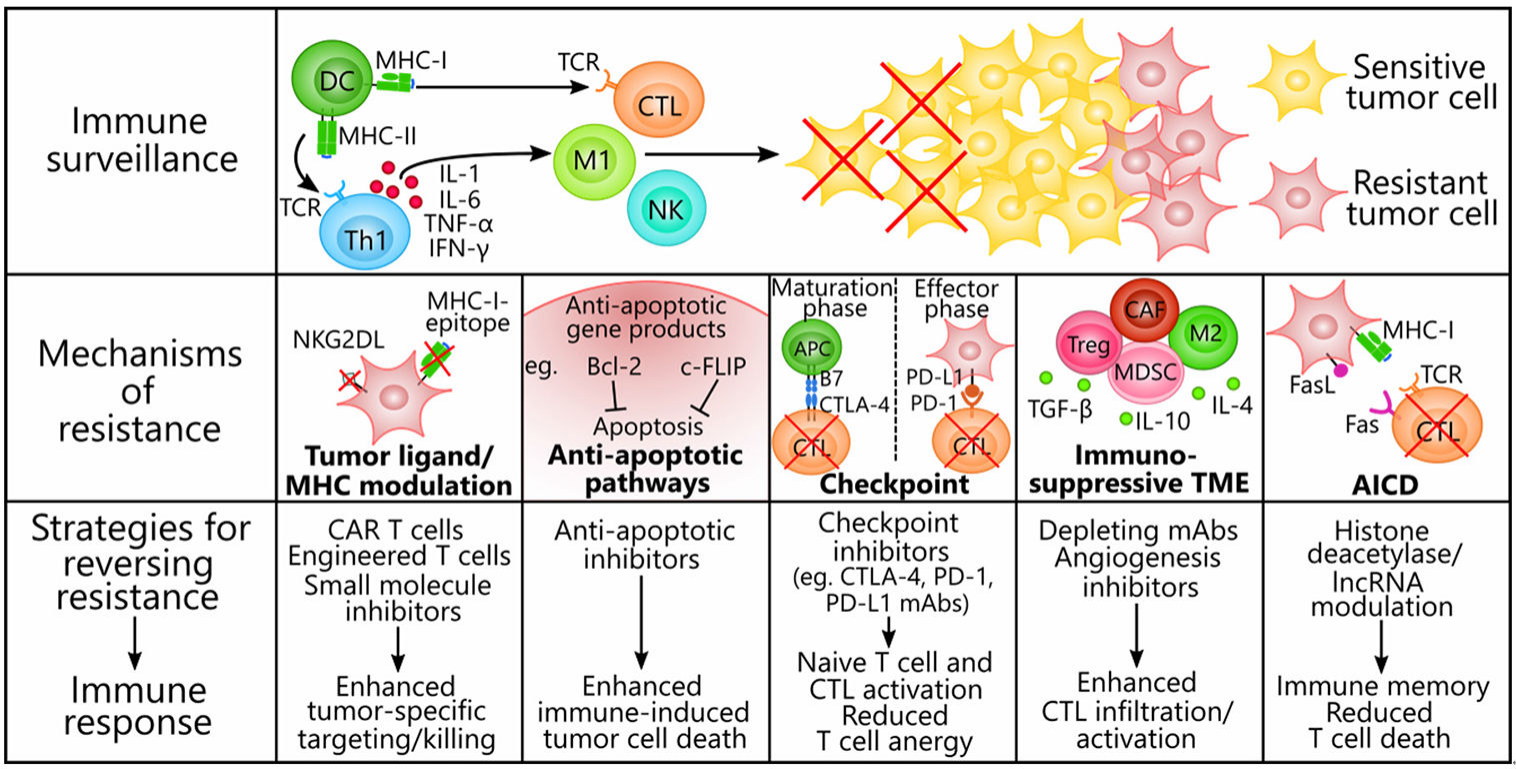
Mechanisms of immune resistance in tumor cells and strategies to reverse resistance. Tumors contain genotypically and phenotypically heterogeneous populations of transformed cells that develop resistance to cancer immune surveillance through innate or ongoing processes of selection by immune cells. Both the innate and the adaptive immune systems participate in an anti-tumor response that involves T cell-priming by DCs and Th1 cytokines (IL-1, IL-6, TNF-α, and IFN-γ) secreted by Th1 cells. Immune effectors infiltrating the tumor, such as CTLs, M1 macrophages, and NK cells, actively eliminate sensitive tumor cells through ADCC, phagocytosis, perforin/granzyme, or TRAIL-mediated apoptosis, whereas resistant tumor cells remain untouched and proliferate. We present five exemplary mechanisms of immune resistance and the corresponding therapies that have proven effective at reversing the alterations responsible for resistance. (1) Tumor cells modulate their expression of immunogenic ligands (e.g., NKG2DLs) and MHC class I molecules to escape detection by CTLs and NK cells. CAR T cells, engineered T cells, and small molecule inhibitors (e.g., MAPK pathway inhibitors) evoke enhanced anti-tumor activity either by acting independently of MHC class I molecules or re-upregulating tumor ligand expression. (2) Tumor cells coopt survival pathways and the anti-apoptotic proteins Bcl-2 and c-FLIP to resist immunogenic cell death via TRAIL or immune cell targeting. Anti-apoptotic inhibitors have proven effective at inhibiting these proteins and reversing the apoptosis-resistant phenotypes. (3) Immune checkpoint ligands (e.g., PD-L1 on tumor cell surfaces and CTLA-4 and PD-1 on T cell surfaces) effectively decommission anti-tumor T cells during the maturation phase of naïve T cells and during the effector/tumor-killing phase. Checkpoint inhibitors are monoclonal antibodies that can synergize with T cell-focused therapies by blocking either the coinhibitory ligand or its receptor to prevent anergy in naïve and mature T cells. (4) The dynamic TME consists of immunosuppressive immune cells (e.g., Treg cells, MDSCs, M2 macrophages, and CAFs) and factors (e.g., TGF-β, IL-10, and IL-4) that suppress anti-tumor effector cell functions and seclude them away from the tumor. Monoclonal antibodies targeted to immunosuppressive cell markers to deplete these cells or angiogenesis inhibitors that recondition the TME are able to enhance CTL activation and infiltration into the tumor. (5) AICD threatens the long-term efficacy of adoptive cell transfer therapies by depleting T cells via Fas-FasL or repetitive TCR stimulation. Previous evidence suggests that halting the conduction of the death signals in T cells by downregulating lncRNA and histone deacetylase expression can inhibit AICD and potentiate the formation of immune memory. AICD: activation-induced cell death; Bcl-2: B cell lymphoma 2; CAR: chimeric antigen receptor; c-FLIP: cellular FLICE (FADD-like IL-1β-converting enzyme)-inhibitory protein; CAF: cancer-associated fibroblasts; CTL: cytotoxic T lymphocyte (CD8+); CTLA-4: cytotoxic lymphocyte-associated protein 4; DC: dendritic cell; FasL: fas ligand; IFN-γ: interferon γ; IL-1: interleukin 1; IL-6: interleukin 6; M1: M1 macrophage; M2: M2 macrophage; MAPK: mitogen-activated protein kinase; MDSC: myeloid-derived suppressor cell; MHC-I: major histocompatibility complex class I; MHC-II: major histocompatibility complex class II; NK: natural killer cell; NKG2DL: natural killer group 2D ligand; PD-1: programmed cell death protein 1; PD-L1: programmed death-ligand 1; TCR: T cell receptor; TGF-β: transforming growth factor-β; Th1: T helper 1 cell (CD4+); TME: tumor microenvironment; TNF-α: tumor necrosis factor α; TRAIL: tumor necrosis factor-related apoptosis-inducing ligand; Treg: T regulatory cell; ADCC: antibody-dependent cell cytotoxicity; MDSCs: myeloid-derived suppressor cells; lncRNA: long non-coding RNA

We briefly describe the results of a few preclinical and early phase clinical trials that have made notable strides in inducing tumor regression and reverting the immunosuppressive phenotype (see the schematic diagram in Figure 2). Currently, several clinical trials are underway in testing the efficacy and safety of CAR T cells, checkpoint inhibitors, and small molecule inhibitors in the treatment of a spectrum of primary and advanced tumors. Several of these drugs have been approved in the US as first or second line therapies^[176]^, although the critical next step is to investigate their efficacy in cancers with scarce treatment options. For example, CAR T cells have not been reliable against solid tumors, as both physical and immunological barriers in the TME diminish the efficacy of the transferred cells^[177]^. However, newly-engineered receptors and reformulations of the CAR T cell machinery may circumvent these barriers when combined with additional agents that can prevent T cell exhaustion and reprogram the TME.

## FUTURE PERSPECTIVES

In addition to those discussed in this review, other novel immune cell-mediated therapies deserve recognition for their potential in anti-cancer treatment. DC vaccines composed of *ex vivo*-generated DCs loaded with tumor-specific antigens can be reinfused into cancer patients to incite a targeted T cell response^[178]^. Pro-inflammatory cytokine therapy can be used to reverse NK cell anergy in MHC-deficient tumors^[179]^. Clinical trials using genetically engineered CAR-NK cells have emerged over recent years, testing the efficacy of an antigen-specific NK cell response that may be a safer alternative to CAR T cell therapy^[15,16,180]^.

The commensal gut microbiota plays an important role in stimulating the host immune system, for example, by producing multiprotein complexes called inflammasomes^[181]^. Researchers are characterizing the patient gut microbiome to link the composition to tumorigenic potential as well as to patient response to anti-PD-1 immunotherapy^[3,181,182]^.

Data generated from high-throughput techniques have made bioinformatics and computational sciences a necessity in translational cancer research^[183]^. The value of biomarkers and stromal gene signatures in predicting treatment response is well established^[121,149]^, and new approaches are developed every year to enhance the power of such strategies in different treatment settings^[184]^.

## CONCLUSION

A large proportion of cancer patients experience little to no benefit from immunotherapies due to overly aggressive tumors or large tumor burdens. As briefly covered above, the phenomenon of clinical cancers refractory to immunotherapies is dependent upon heterogeneity within the tumor, the innate and adaptive responses, and the TME. Extrinsic pressure from immune surveillance causes tumor cells that survive the onslaught of apoptotic signals to alter their antigenic landscape and mold the microenvironment to their liking. By releasing immunosuppressive cytokines and chemoattractants, they recruit stromal cells, including T_reg_ cells, M2 macrophages, and MDSCs, which exhaust existing lymphocytes and skew the response from an anti-tumor Th1 to a pro-tumorigenic Th2 response. Advancements in immunotherapy have countered these tumor adaptations with therapies that have enhanced specificity of attack, blocked checkpoint-induced exhaustion, downregulated anti-apoptotic pathways, and reversed the immune tolerant TME, with any combination of those mentioned. A cursory glance at immunotherapies beyond those dependent on CTLs and tumor intrinsic pathways revealed a focus on the supportive, antigen-presenting cells of the immune system and holistic approaches to anti-cancer therapies. As we venture toward new and improved immunotherapies, we will inevitably encounter a range of response rates, even with identical tumor types among patients. Thus, it is essential to consider a broad perspective of the field to take measured steps toward both preventing and reversing immune resistance.
